# Surveillance of device associated infections and mortality in a major intensive care unit in the Republic of Cyprus

**DOI:** 10.1186/s12879-017-2704-2

**Published:** 2017-09-06

**Authors:** Stelios Iordanou, Nicos Middleton, Elizabeth Papathanassoglou, Vasilios Raftopoulos

**Affiliations:** 1 Nursing Department, General Hospital of Limassol, Cyprus University of Technology, Limassol, Cyprus; 2 Nursing Department, Cyprus University of Technology, 15, Vragadinou Str, 3041 Limassol, Cyprus; 3 Faculty of Nursing, University of Alberta, Edmonton, Alberta Canada

**Keywords:** Healthcare-associated infection, Device associated infection, Intensive care unit, Ventilator associated pneumonia, Central line-associated blood stream infection, Catheter-associated urinary tract infection

## Abstract

**Background:**

Device-associated health care-associated infections (DA-HAI) pose a threat to patient safety, particularly in the intensive care unit. The aim of this study was to assess the incidence of DA-HAIs, mortality and crude excess mortality at a General Hospital’s Intensive Care Unit (ICU) in the Republic of Cyprus for 1 year period.

**Methods:**

A prospective cohort, active DA-HAIs surveillance study with the use of Health Acquired Infections (HAIs) ICU Protocol (v1.01 standard edition) as provided by ECDC/NHSN for the active DA-HAIs surveillance study was conducted. The study sample included 198 ICU patients admitted during the research period and hospitalized for over 48 h. The Ventilator-Associated Pneumonia (VAP), Central Line-Associated Bloodstream Infection (CLABSI), and Catheter-Associated Urinary Tract Infection (CAUTI) rates, length of stay (LOS), mortality, and crude excess mortality were calculated.

**Results:**

CLABSI was the most frequent DA-HAI with 15.9 incidence rate per 1000 Central Venus Catheter (CVC) days. The VAP rate, was 10.1 per 1000 ventilator days and the CAUTI rate was 2.7 per 1000 urinary catheter days. Device associated infections were found to be significantly associated with the length of ICU stay (*p* < 0.001), the CVC days (*p* < 0.001), ventilator days (*p* < 0.001), and urinary catheter days (*p* < 0.001). The excess mortality was 22.1% for those who acquired a DA-HAI (95% CI, 2–42.2%) compared to the patients who remained DA-HAI free. Mortality of patients with VAP infection was 2.3 times higher (RR = 2.33 95% CI, 1.07–5.05) than those patients admitted without a HAI and subsequently did not acquire a DA-HAI. The most frequently isolated pathogen was *Staphylococcus epidermidis* (13.9%) and *Candida albicans* (13.9%).

**Conclusions:**

Higher DA-HAIs rates and device utilization than the international benchmarks were found in this study, calling into question the safety of preventative practices employed in this unit.

## Background

Device-associated health care–associated infections (DA-HAIs) affect the quality of health care in terms of increased morbidity, mortality, and additional cost for patient care provision. DA-HAIs pose a severe threat to patients, despite prevention efforts that have resulted to a significant decrease of infections’ incidence [1–5].

CLABSIs, VAPs and CAUTIs are the three most common DA-HAIs [6]. The incidence of DA-HAIs in the European Union (EU) countries varies. VAP median rate in 11 major EU countries, reported by ECDC 2014 annual epidemiology report, was 8.4 per 1000 ventilator days (range: 3.9–14.3/1000 device days) with the lowest rates to be reported in Luxemburg (3.2 [2.7–3.9]/1000 device days) and the highest in France (12.8 [8.4–18.3]/1000 device days) [7]. The median rate of CLABSIs among ten EU countries was 1.5 per 1000 central vein catheter (CVC) days (0.93–3.27/1000 CVC days) [8] and for CAUTI’s among 23 EU countries was 1.3 per 1000 urine catheter days (1.2–1.5/1000 CVC days).

The current study is assessing the incidence of DA-HAIs, mortality and crude excess mortality of a closed type, General Hospital’s ICU in the Republic of Cyprus for 1 year period and reporting the results of the active DA-HAIs surveillance system as a part of a comprehensive prevention program.

## Methods

### Setting

The study was conducted in the ICU of a major public secondary general referral hospital in the Republic of Cyprus with 28.000 yearly hospital admissions. Although this is an adult hospital, pediatric patients can be admitted to the pediatric department but not in the ICU.

The unit is a closed adult ICU, of open plan, case mixed, with eight beds. It serves primarily the south area of the island; however, patients may be admitted from the private and other public hospitals across the Republic of Cyprus. During the day time shift, the staff consists at least of six nurses (≈0.75 nurse per bed) and five ICU physicians (intentivists) in the morning, whereas during the afternoon and night shift the staff consists of four nurses (one nurse for every two beds) and one physician.

### Study design and data collection

A prospective cohort, active DA-HAIs surveillance study was conducted using standardized survey record form for collecting patients’ data, based on the ICU protocol (ECDC-NHSN, HAI-ICU Protocol, v1.01 standard edition) [9] for a period of 12 months (January–December, 2015). The study protocol was approved by the Cyprus Bioethics Committee (EEKB/ΕΠ/2015/37) and reviewed by the Republic of Cyprus Personal Data Commissioner.

### Patients

All the patients admitted to the ICU and hospitalized for more than 48 h (*n* = 198) were included in the study. They were monitored for DA-HAIs, until their death or discharge from the ICU. Patients’ demographics, acute chronic health evaluation (APACHE II) [10], simplified acute physiology score (SAPS II) [11], date and site of DA-HAIs onset, duration of device usage (days), isolated pathogens, antibiogram results, length of patient stay and patient outcome on discharge from ICU were recorded.

The data were collected by experienced ICU nurses and physicians, who had attended a short-term training session regarding the DA-HAIs diagnostic criteria and ECDC-NHSN protocol. Although that infection control committees, have been in operation since 1999, the vast majority of the committee members are not specialized or accredited in infection control.

### Sampling and laboratory testing

All the patients admitted to the ICU were screened to detect multidrug-resistant organisms (MDROs) colonization using an admission surveillance culture protocol (nasal-rectal samples).

Blood samples were collected in case of a suspected blood stream infection. For CLABSIs, the CVC was aseptically removed and the distal 4 cm of the catheter was separated and cultured. For CAUTIs, urine samples were collected by aseptically aspirating a sample from the urine sample port. Quantitative culture for aerobic bacteria was performed using samples of lower respiratory tract secretions to detect VAP. Lower respiratory tract secretions were collected using tracheal aspiration or/and broncheoalveolar lavage (BAL).

Standard laboratory methods were used to identify microorganisms using automated method Phoenix 100 and Vitek II.

### DA-HAIs rate calculations

For estimating DA-HAIs incidence density rates, confirmed VAP events were divided by the number of total ventilator days and multiplied by 1000. For CLABSI, the confirmed CLABSI events were divided by total CVC days multiplied by 1000 and for CAUTI, the confirmed CAUTI events were divided by total Urine Catheter (UC) days multiplied by 1000. Device utilization ratios have been calculated by dividing the total number of device-days by the total number of patient-days. Device-days are the total number of days of exposure to each device (ET, CVC, or UC) for all the patients during the selected time period. Patient-days are the total number of days that patients are in the ICU during the selected time period.

### Crude excess mortality

The mortality of patients with a DA-HAI was compared with the mortality of HAI-free patients (who did not acquire a DA-HAI during their stay in the ICU) by calculating and evaluating crude excess mortality. Crude excess mortality in the ICU was defined as the difference between the crude overall case fatality rate of patients with a DA-HAI and that of patients admitted without a HAI who did not acquire a DA-HAI in the ICU during the same period.

### Statistical analysis

Medians and interquartile ranges (IQR) were used to describe the distribution of continuous variables and frequencies and percentages for categorical variables. Comparisons between the two groups were performed by using the Mann-Whitney U test for continuous variables, and the Fisher exact test or the chi-square test for categorical variables. Relative risk has been calculated for comparison of mortality rates for patients with a DA-HAI versus mortality rate for patients admitted without an HAI who did not acquire a DA-HAI. Relative Risks (RRs) were calculated using a bionomial regression with a log link function adjusted by Age. Both unadjusted and adjusted RRs are reported. Descriptive statistics, correlation tests and relative risks were calculated in the IBM-SPSS software, version 21. Moreover, 95% confidence intervals of the incident rates and excess mortality were calculated in R version 3.1.3 [12] using the packages exactci [13] for the incident rates, and PropCIs [14] for the excess mortality.

## Results

During the study period, surveillance data were collected for 198 (73 females [36.9%] & 125 males [63.1%]) patients hospitalized in the ICU for a total of 2269 ICU days.

Median age was 68 years old (IQR, 55–77). The median APACHE II score on admission was 22 (IQR, 16–28), whereas the median SAPS II score was 49 (IQR, 36–65). Median length of ICU stay was 6 days (IQR, 4–13). The median time interval between admission and identification of the first DA-HAI was 5 days (IQR, 4–10).

One hundred and fifty-one of the 173 patients were admitted without an HAI and did not acquire a DA-HAI during their stay (Table 1).

**Table 1:** Baseline characteristics across patients who acquired a DA-HAI

Characteristic	Patients without infection (*n* = 173)		Patients with infection (ν = 25)			All patients (*n* = 198)
	*N* (%)	Median (IQR)	*N* (%)	Median (IQR)	*p*-value†	
Gender						
Female	67 (38.7)		6 (24.0)		0.187	73 (36.9)
Male	106 (61.3)		19 (76.0)			125 (63.1)
Age in years		68 (56–79)		67 (49–70)	0.081	68 (55–77)
Days of ICU stay		6 (4–9)		31 (22–40)	<0.001	6 (4–13)
Apache II score		22 (16–28)		25 (18–29)	0.294	16 (22–28)
SAPS II		49 (36–65)		48 (38–66)	0.625	49 (36–65)
Type of admision						
medical	124 (71.7)		17 (68.0)		0.701	141 (71.2)
schedule surgical	3 (1.7)		0 (0.0)			3 (1.5)
Unschedule surgical	46 (26.6)		8 (32.0)			54 (27.3)
Origin of patient						
Community	60 (34.7)		3 (12.0)		0.004	63 (31.8)
LTCF^a^	2 (1.2)		2 (8.0)			4 (2)
other ICU	33 (19.1)		10 (40.0)			43 (21.7)
Ward this or other hospital	78 (45.1)		10 (40.0)			88 (44.4)
Trauma	22 (12.7)		5 (20.0)		0.349	27 (13.6)
Impaired immunity	31 (17.9)		7 (28.0)		0.276	38 (19.2)
Antimicrobial treatment	156 (90.2)		24 (96.0)		0.48	180 (90.9)
Acute Coronary care	41 (23.7)		8 (32.0)		0.456	49 (24.7)
Central Vascular Catheter DAYS		6 (4–10)		15.5 (9–33)	<0.001	7 (4–12)
Mechanical Ventilation DAYS		5 (2–8)		23 (10–39)	<0.001	6 (3–10)
Urinary catheter DAYS		6 (4–9)		31 (22–40)	<0.001	6.5 (4–13)

†Chi-square test for type of admission and origin of patients. Fisher’s exact test for the rest of the categorical variables. Man-Whitney U test for the scale variables (e.g. Age. Days in ICU. etc.)

^a^Long Term Care Facility

A total of 43 instances of DA-HAIs were detected in 25 of the 198 patients, indicating an overall infection rate of 12.6% or an overall incidence of 19 DA-HAIs per 1000 ICU-days (95%[CI:13.7–25.5]). CLABSI (48.8%, 15.9/1000 device days) was the most commonly encountered type of infection accounting for 48.8% of all the DA-HAIs, followed by VAP (37.2%, 10.1/1000 device days) and CAUTI (14%, 2.7/1000 device days). The proportion of patients exposed to mechanical ventilation was 70%, those exposed to central catheters were 58% and those to urinary catheters 99% (device utilization 0.70, 0.58 and 0.99 for mechanical ventilation, central catheters and urinary catheters respectively) (Table 2).

**Table 2:** Type and incidence of DA-HAIs

	Device-days	Device use	No. of infections	% of infections	Incidence per 100 patients	Incidence per 1000 device-days	Exact 95 CI	
VAP	1584	0.70	16	37.2	8.08	10.10	5.8	16.4
CLABSI	1318	0.58	21	48.8	10.61	15.93	9.9	24.3
CAUTI	2259	0.99	6	14.0	3.03	2.66	1	5.8

*CAUTI* catheter-associated urinary tract infection, *CLABSI* central catheter-associated bloodstream infection, *VAP* ventilator-associated pneumonia

Patients who did not acquire a DA-HAI, had a median ICU stay of 6 days (IQR, 4–9), while patients who acquired at least one DA-HAI had a median ICU stay of 31 days (IQR, 22–40). The crude ICU mortality was 40% (10/25) for the patients who acquired a DA-HAI and 17.9% (31/173) for those without a DA-HAI, yielding an overall excess mortality of 22.1% (95% CI, 4.2–42.2%). The multivariate binomial regression model adjusted for age, showed that the mortality rate of patients who acquaired a DAI, was 2.65 times higher (RR = 2.65 95% C.I.(1.48–4.72) than that of the patients who did not acquire a DAI during their stay.

The crude ICU mortality rate (Table 3) was 16.6% for patients admitted without an HAI who did not acquire a DA-HAI in the ICU. The crude ICU mortality rate for patients with VAP (38.5%) is 2.3 times higher (RR = 2.33 95% C.I.(1.07–5.05) than the crude mortality rate for patients without HAI on admission, who did not acquire a DA-HAI in the ICU (16.7%), yielding an overall crude excess mortality rate of 21.9%. When adjusting for the age of the patient, the RR of mortality of VAP patients over the nonHAI/nonDAI, is increased to 3.62 (95% C.I.(1.64–7.98).

**Table 3:** Mortality rates for DA-HAIs

		Mortality			Relative Risk		
	Total patients	Crude		Crude excess		95 CI	
		*N*			RR	Lower	Upper
None^a^	151	25	16.6				
VAP	13	5	38.5	21.8	2.33	1.07	5.05
CLABSI	15	5	33.3	16.7	2.01	0.90	4.48
CAUTI	6	2	33.3	16.7	2.01	0.61	6.58

^a^Patients admitted with no HAI and acquired no HAI no DA-HAI. Table presents Risk Ratios (RRs) unadjusted of any demographic characteristics. The multivariate model adjusting for Age revieled the following RRs: VAP:RR = 3.62 95% C.I.(1.64–7.98), CLABSI:RR = 2.32 95% C.I.(1.04–5.15), CAUTI:RR = 2.37 95% C.I.(0.73–7.74*)*

### Microorganisms’ profile and antimicrobial resistance

The most frequently isolated pathogens, were *Staphylococcus epidermidis* (13.9%), *Candida albicans* (13.9%), Pseudomonas Aeruginosa (11.6%), followed by Stenotrophomonas Maltophilia (7%), *Escherichia coli* (7%) and Acinetobacter Baumannii (7%). *Staphylococcus aureus* was detected in 2 DAIs (4.6%).

Pseudomonas Aeruginosa (*n* = 4/25%), *Candida albicans* (*n* = 4/25%), and Acinetobacter Baumannii (*n* = 2/12.5%) were most prevalent in the 16 cases of VAP. *Staphylococcus epidermidis* (*n* = 6/25.6%) was most prevalent in the 21 cases of CLABSI, followed by Stenotrophomonas Maltophilia, Staphylococcus Haemolyticus, other coagulase-negative staphylococci and *Escherichia coli* with 2 cases each (9.5%).

*Candida albicans* (*n* = 2/33.3%) was most prevalent in CAUTI infections. *Candida tropicalis*, Enterobacter Gergoviae, *Escherichia coli* and Pseudomonas Aeruginosa microbes appeared once (Table 4).

**Table 4:** Pathogen prevalence out of the 43 DAIs

ECDC CODE	PATHOGEN		NUMBER
STAEPI	*Staphylococcus epidermidis*	13.95	6
CANALB	*Candida albicans*	13.95	6
PSAER	Pseudomonas aeruginosa	11.63	5
STEMAL	Stenotrophomonas maltophilia	6.98	3
ESCCOL	*Escherichia coli*	6.98	3
ACIBAU	Acinetobacter baumannii	6.98	3
ENCFAE	Enterococcus faecalis	4.65	2
STAHAE	Staphylococcus haemolyticus	4.65	2
STAOTH	Other coagulase-negative staphylococci	4.65	2
SERMAR	Serratia marcescens	4.65	2
CANKRU	*Candida krusei*	2.33	1
STAAUR	*Staphylococcus aureus*	2.33	1
CANTRO	*Candida tropicalis*	2.33	1
ENBCLO	*Enterobacter cloacae*	2.33	1
STANSP	Staphylococcus spp.. not specified	2.33	1
CANPAR	*Candida parapsilosis*	2.33	1
ENBGER	Enterobacter gergoviae	2.33	1
SERLIQ	Serratia liquefaciens	2.33	1
KLEOTH	Klebsiella species. (Non oxytoca. Non pneumoniae)	2.33	1
Total		100.00	43

### Resistance

The two cases of *Staphylococcus aureus* were MRSA (oxacillin resistant) but sensitive to Glycopeptides (vancomycin, teicoplanin). All three cases of Acinetobacter Baumannii (*n* = 3/100%) were resistant to carbapenems (imipenem, meropenem, doripenem – not ertapenem) and collistin. Out of the 5 cases of Pseudomonas Aeruginosa, 5 (100%) were Ceftazidim sensitive, 3 (60%) sensitive to Piperacillin or Ticarcillin with or without enzyme inhibitor and 3 (60%) sensitive to carbapenems (imipenem, meropenem, doripenem – not ertapenem). Two cases (40%) were resistant to Piperacillin or Ticarcillin and to carbapenems one case (20%) was resistant to collistin.

There were eight cases of Enterobacteriaceae with pathogens which were found to be Serratia Marcescens (2) Klebsiella species. (non oxytoca, non pneumoniae) (1), Enterobacter gergoviae (1), *Escherichia coli* (2) and *Enterobacter cloacae* (2). These cases were sensitive to carbapenems (6/75%), third generation cephalosporins (cefotaxime, cetriaxone, ceftazidime) (5/62.5%) and amoxicillin/clavulanate (3/37.5%). Enterobacteriaceae resistance was found on carbapenems (25%), third generation cephalosporins (3/37.5%) and amikacin (5/62.5%). Extended beta-lactamase producer, was found on 3 (37.5%) of the Enterobacteriaceae.

## Discussion

This study reports the results of the first active DA-HAIs surveillance study that has been conducted in an ICU of the southern part of the Republic of Cyprus. The overall DA-HAIs rate is 12.62% or 19 DA-HAIs per 1000 ICU days. Even though our rates are significantly lower than the rates reported in other studies [15–20], our findings suggest that such rates have an impact on the length of stay (LOS) and mortality rate. Patient with DA-HAIs had a median LOS longer than patients without DAI-HAIs. The overall excess mortality of the patients who acquired DA-HAI (22.1%) was lower than the previous Cypriot report [20] (33.2%) and twice higher than that reported at the International Nosocomial Infection Control Consortium in Lebanon (INICC-2012) (9.8%) [21].

Utilization rates were found to be 70, 58 and 99.6% for mechanical ventilation (MV), central catheters (CVC) and urinary catheters (UC) respectively. The findings of the study for MV and CVC were significantly lower than INICC [22] 2009–2015 (74, 104 and 82% respectively) and Greek report for 2013 [15] (95%, 94% respectively), but all were much higher than INICC 2004–2009 [23], INICC 2007–2012 [24] and other studies [25]. Utilization rates and severity scores must be examined together in order to reflect the severity index of the patients that were admitted to the ICU, since the increased device utilization may be a result of disease severity.

In our study, the median for APACHE II and SAPS II scores for patients with and without DA-HAI were found to be 22 vs 25 and 49 vs 48 respectively. There was not any significant correlation between the two scoring systems or the DA-HAI positive and negative groups. However, predicted mortality rate (PMR) for APACHE II [26] (22 to 25 score) was 40–55% and for SAPS II [11] (48–49 score) 50%. PMR calculations using APACHE II score, indicates that patients’ health condition severity in our study is higher than reported for New York (2004–2007) [27], higher than the Greek report observations for 2013 (16 [score]/15% [PMR]) [15] and similar to Haq et al. 2014 [28]. Comparisons between the similar reported severity scores studies [15, 27] and their device utilization rate showed large deviation. This probably means that severity scores cannot estimate or be a sole drive factor for device utilization use. Overall extended device utilization rates in our findings require further investigation.

The most commonly encountered infection was CLABSI (48.8%) followed by VAP (37.2%) and CAUTI (14%). The hierarchical distribution of DA-HAIs, CLABSI and VAP rates appears to be similar [15, 20, 29–31] or different [22, 32] with the current study pattern, but in general ICU CAUTI rates are found to be fewer in most reports [15, 20, 22, 29, 31, 33].

Incidence density rates for VAP, CLABSI and CAUTI were 10.1, 15.9 and 2.7 per 1000 device-days respectively. In comparison with the findings of the previous Cypriot report [20] higher VAP density rates were found (10.1 vs 6.4). Rates were the same for CAUTI (2.66 vs 2.8) but slightly lower rates for CLABSI (15.9 vs 17.9) were observed [15] for VAP, slightly lower for CAUTI (10.1 vs 20, 2.66 vs 4.3 respectively) and higher for CLABSI (15.9 vs 11.8). Our findings showed lower rates compared to the INICC Malaysia report 2016 [22] for VAP (10.1 vs 21) and CAUTI (2.66 vs 5) and higher CLABSI (15.9 vs 9.4). Overall, DA-HAIs rates results are much higher than the participating NHSN report [34] ICUs: for VAP, 10.1 versus 4; for CLABSI, 15.9 versus 3 and lower for CAUTI, 2.66 versus 3.2 infections per 1000 device-days. These findings lead us to the conclusion that a comprehensive program for reducing VAP and CLABSI is a necessity in this ICU.

A crude ICU mortality rate of 40% for the patients who acquired DA-HAI and 17.9% for patients who did not, was observed. These findings are higher than INICC for Cuba [35] and Greece [15] but similar to the previous Cypriot [20] report (33, 31.2 and 42.6% respectively). The higher rates of our study may reflect a higher severity of underlying disease, since the APACHE II and SAPS II scores are higher than some of the comparison studies.

Crude excess mortality for VAP (21.9%) was higher than CLABSI (16.7%) and CAUTI (16.7%), and higher than data reported by the INICC 2004–2009 [23] (15.2%), INICC 2009–2015 [22] (14.8%), an Egyptian study [36] (12.9%) and Greece (13.0%). CLABSI crude excess mortality (16.7%) was found to be three times lower than the Egyptian study report (45.7%), similar to INICC 2004–2009 report (14.7%) [23] and lower than the Greek report (20.6%) [15]. CAUTI excess mortality was found to be similar to CLABSI (16.7%) in the results of the current study, lower than Greek report [15] (18.7%) and significantly lower than INICC 2009–2015 [22] (32.2%). Overall VAP crude excess mortality is shown as higher and CLABSI and CAUTI similar to or lower than the international benchmarks.

Compared to EU ICU data, the proportion of Pseudomonas aeruginosa isolation (11.63%) was lower than most of the EU countries, similar to Germany and twice higher than United Kingdom, *Candida albicans* (13.95%) lower than Austria and higher than the other 12 and Stenotrophomonas maltophilia (6.98%) lower then Luxemburg and higher than the rest. In general all the proportions of isolation pathogens found in this study were higher than the majority of the reported pathogens proportion in ICUs across EU [7]. Unexpectedly high was the proportion of *Staphylococcus epidermidis* isolation (13.9%). This finding may reflect problems in sample collection procedure or diagnosis.

These results will be presented to the staff, in order to create the sense of urgency followed by an intervention that includes the implementation of a comprehensive care-bundle approach program for infection surveillance, prevention and control in order to improve their compliance with standard precautions and devices maintenance in the current ICU.

### Limitations

The current study has certain limitations. The study was conducted in one of the only two closed type, adult ICUs in Cyprus. These results cannot be generalized to other public or private hospital settings. DA-HAIs cost estimation was one of the study limitations due to the lack of financial reports.

Despite the study’s limitations, it can provide clinicians with valuable data with regard to incidence rates and prevalence of DA-HAIs as data for the Republic of Cyprus cannot be found easily in the published literature due to the lack of a Cypriot infection surveillance system.

## Conclusions

In this study, the high incidence rates of DA-HAIs prevalence, device utilization and the antimicrobial resistance pattern, were found to emphasize the need to implement a comprehensive care-bundle approach program. A national active infection surveillance system can be set up to improve current infection control, data collection practices as well as changes in device usage and their maintenance.

## Abbreviations

CAUTI: Catheter-associated urinary tract infection
CLABSI: Central line-associated blood stream infection
CVC: Central venus catheter
DA-HAI: Device-associated health care-associated infections
ICU: Intensive care unit
VAP: Ventilator associated pneumonia
